# Introduction to Translational Bioinformatics Collection

**DOI:** 10.1371/journal.pcbi.1002796

**Published:** 2012-12-27

**Authors:** Russ B. Altman

**Affiliations:** 1 Department of Genetics, Stanford University, Stanford, California, United States of America; Whitehead Institute, United States of America; University of Maryland, Baltimore County, United States of America

This article is part of the “Translational Bioinformatics” collection for *PLOS Computational Biology*.

How should we define translational bioinformatics? I had to answer this question unambiguously in March 2008 when I was asked to deliver a review of “recent progress in translational bioinformatics” at the American Medical Informatics Association's Summit on Translational Bioinformatics. The lecture required me to define papers in the field, and then highlight exciting progress that occurred over the previous ∼12 months. I have repeated this for the last few years, and the most difficult part of the exercise is limiting my review only to those papers that are within the field.

I have never worried much about definitions within informatics fields; they tend to overlap, merge and evolve. “Informatics” seems clear: the study of how to represent, store, search, retrieve and analyze information. The adjectives in front of “informatics” vary but also tend to make sense: medical informatics concerns medical information, bioinformatics concerns basic biological information, clinical informatics focuses on the clinical delivery part of medical informatics, biomedical informatics merges bioinformatics and medical informatics, imaging informatics focuses on…images, and so on. So what does this adjective “translational” denote?

Translational medical research has emerged as an important theme in the last decade. Starting with top-down leadership from the National Institutes of Health and its former Director, Dr. Elias Zerhouni, and moving through academic medical centers, research institutes and industrial research and development efforts, there has been interest in more effectively moving the discoveries and innovations in the laboratory to the bedside, leading to improved diagnosis, prognosis, and treatment. Translational research encompasses many activities including the creation of medical devices, molecular diagnostics, small molecule therapeutics, biological therapeutics, vaccines, and others. One of the main targets of translation, however, is revolutionary explosion of knowledge in molecular biology, genetics, and genomics. Some believe that the tremendous progress in discovery over the last 50+ years since elucidation of the double helix structure has not translated (there's that word!) into much practical health benefit. While the accuracy of this claim can be debated, there can be no debate that our ability to measure (1) DNA sequence (including entire genomes!), (2) RNA sequence and expression, (3) protein sequence, structure, expression and modification, and (4) small molecule metabolite structure, presence, and quantity has advanced rapidly and enables us to imagine fantastic new technologies in pursuit of human health.

There are many barriers to translating our molecular understanding into technologies that impact patients. These include understanding health market size and forces, the regulatory milieu, how to harden the technology for routine use, and how to navigate an increasingly complex intellectual property landscape. But before those activities can begin, we must overcome an even more fundamental barrier: connecting the stuff of molecular biology to the clinical world. Molecular and cellular biology studies genes, DNA, RNA messengers, microRNAs, proteins, signaling molecules and their cascades, metabolites, cellular communication processes and cellular organization. These data are freely available in valuable resources such as Genbank (http://www.ncbi.nlm.nih.gov/genbank/), Gene Expression Omnibus (http://www.ncbi.nlm.nih.gov/geo/), Protein Data Bank (http://www.wwpdb.org/), KEGG (http://www.genome.jp/kegg/), MetaCyc (http://metacyc.org/), Reactome (http://www.reactome.org), and many other resources. The clinical world studies diseases, signs, symptoms, drugs, patients, clinical laboratory measurements, and clinical images. The emergence of clinical and health information technologies has begun to make these clinical data available for research through biobanks, electronic medical records, FDA resources about drug labels and adverse events, and claims data. Therefore, a major challenge for translational medicine is to connect the molecular/cellular world with the clinical world. The published literature, available in PubMED (http://www.ncbi.nlm.nih.gov/pubmed), does this, as does the Unified Medical Language System (UMLS) that provides a *lingua franca* (http://www.nlm.nih.gov/research/umls/). However, it falls to translational bioinformatics to engineer the tools that link molecular/cellular entities and clinical entities. Thus, I define “translational bioinformatics” research as the development and application of informatics methods that connect molecular entities to clinical entities.

In this collection, Dr. Kann and colleagues have assembled a wonderful group of authors to introduce the key threads of translational bioinformatics to those new to the field. The collection first provides a conceptual overview of the key data and concepts in the field, and then introduces some of the key methods for informatics discovery and applications. Just by examining the table of contents on the collection page (http://www.ploscollections.org/translationalbioinformatics), it is clear that many exciting and emerging health topics are squarely within the scope of translational bioinformatics: cancer, pharmacogenomics, medical genetics, small molecule drugs, and diseases of protein malfunction. There is an unmistakable flavor of personalized medicine here as well (genome association studies, mining genetic markers, personal genomic data analysis, data mining of electronic records): our molecular and clinical data resources are now allowing us to consider individual variations, and not simply population averages. I congratulate the editors and authors on creating an important collection of articles, and welcome the reader to an exciting field whose challenges and promise are unbounded.

